# Constitutional Reform by Legal Transplantation: The United Kingdom Internal Market Act 2020

**DOI:** 10.1093/ojls/gqac018

**Published:** 2022-09-16

**Authors:** Thomas Horsley

**Affiliations:** Reader, School of Law and Social Justice, University of Liverpool

**Keywords:** constitutional law, constitutional theory, devolution, comparative law, judicial review, public law

## Abstract

This article develops the comparative law framework on legal transplantation to theorise the impact of the United Kingdom Internal Market Act 2020 (UKIMA) on the UK constitution across three registers of analysis—the territorial, the material and the conceptual. It arrives at three conclusions. First, in relation to the territorial constitution, this article argues that the UKIMA introduces something transformative: the concept of an internal market as a shared regulatory space that cuts across the respective competences of the UK and devolved legislatures. Secondly, the legal transplant framework points to the introduction of a powerful commitment to the principles of a liberal market economy as the basis of the UK’s material constitution. Finally, regarding the conceptual constitution, this article concludes that the UKIMA effects a qualitative change to established patterns of judicial review through its co-opting of courts as agents to secure the foundations of the newly recast material constitution.

## 1. Introduction

The United Kingdom’s withdrawal from the European Union has precipitated the wholescale integration of a vast body of Union law into the UK legal system. Key provisions of EU law that previously found their place within the domestic legal order by virtue of the European Communities Act 1972 have been directly transposed into UK law through a range of legislative enactments.^1^ EU withdrawal has also triggered the adoption of new legislative frameworks that draw on aspects of Union law to address domestic regulatory challenges.^2^ The repeal of the 1972 Act,^3^ coupled with the imperative to ensure legal stability post-Brexit, necessitated this activity. Paradoxically, the UK’s departure from the EU has resulted in a significant increase in the prevalence of EU norms within the domestic legal order, albeit on a qualitatively different basis.^4^

This article develops the concept of legal transplantation—familiar currency in comparative law scholarship—to assess the implications of this development for the UK constitution.^5^ Theorising the domestication of EU legal norms as legal transplants provides a robust framework to assess the impact of shifting legal norms between distinct legal systems—in this instance, from a quasi-federal supranational ‘new legal order’^6^ to a unitary state structured around a keystone principle of parliamentary sovereignty. As section 2 outlines, the legal transplant framework recognises an important dual dynamic, namely that the movement of norms between legal systems both *determines* and is *determined by* the constitutional conditions that prevail within the receiving legal system.^7^ Existing analyses of the UK constitution engage only implicitly with the second aspect of that dynamic, typically by rationalising examples of legal transplantation with established features of the UK constitution, notably the doctrine of parliamentary sovereignty.^8^ The legal transplant framework developed in this article engages with the dual dynamic explicitly to generate new insights into the UK constitution and processes of domestic constitutional reform.

This article focuses attention on one specific instance of legal transplantation arising as a result of Brexit: the United Kingdom Internal Market Act 2020 (UKIMA). That Act seeks to establish a functioning internal market across the four nations of the UK—an objective previously largely secured by virtue of the UK’s membership of the EU internal market.^9^ Section 3 reviews the provisions of the UKIMA with reference to the EU internal market from which it explicitly draws much of its substance. Reviewing that Act’s key provisions, it points to instances of legal transplantation at three distinct levels of analysis. The parallels between the EU and UK internal markets are—as the legal transplant framework would forecast—not exact, but unmistakable nonetheless.

Having identified the UKIMA as a legal transplant, this article then explores the constitutional implications that follow from that conclusion across three ‘registers’ of analysis. First, section 4 explores the impact of the Act on the *territorial* features of the UK constitution with a particular focus on devolution arrangements. Secondly, section 5 assesses the implications of the UKIMA on the UK’s *material* constitution; in other words, it explores the extent to which that Act reshapes its substantive aims and objectives. Thirdly, section 6 uses the legal transplant framework to theorise the impact of the UKIMA on the *conceptual* foundations of the UK constitution. This involves reflecting on the extent to which the UKIMA affects the nature of the UK constitution under the doctrine of parliamentary sovereignty.

This article arrives at three main conclusions. First, in relation to the territorial constitution, it demonstrates that the UKIMA introduces, through legal transplantation, something new and distinctive: the concept of an internal market as a shared regulatory space that cuts across the respective competences of the UK and devolved administrations. In common with the EU model from which it borrows, that new regulatory space is premised on a federal approach to managing the coexistence of different sites of legislative and administrative authority. At the same time, the adoption and design of the UKIMA evidences the determinative impact of established features of the UK’s territorial constitution on the EU internal market as an object of legal transplantation. What emerges is a regulatory framework that is incomplete, coercive and, mirroring existing constitutional arrangements, highly asymmetrical. Rather than innovate to bring together the UK and devolved governments voluntarily as partners in the management of the UK internal market as a newly constituted shared regulatory space, the UKIMA instead ultimately privileges, by imposition, the institutional role of the UK government (and UK Parliament) in future market-management.

Secondly, in relation to the material constitution, the UKIMA introduces a new domestic legal commitment to the principles of a liberal market economy. That commitment, which the UKIMA transplants from the EU legal order to restrain the exercise of legislative and administrative policy-making post-Brexit, is previously absent from the UK’s domestic constitutional arrangements. It is also qualitatively different from earlier reforms to the UK’s material constitution; for example, the reconceptualisation of civil liberties as fundamental rights under the Human Rights Act 1998 (HRA). The attribution of primacy to economic objectives remains highly controversial in the EU legal order, where it functions, in accordance with the case law of the European Court of Justice (ECJ), to subordinate the protection of non-economic objectives to the demands of market principles.^10^ Transposed to the domestic legal order, the changes that the UKIMA makes to the UK’s material constitution are likely to give rise to parallel tensions as enforceable restraints on democratic majoritarian decision-making. As will be further argued (section 5B), under the doctrine of parliamentary sovereignty, this is first and foremost a challenge for the devolved administrations.

Thirdly, in relation to the conceptual constitution, as a legal transplant, the UKIMA contributes to the further judicialisation of the UK constitution through its establishment of new judicial review competences. Its introduction of the market access principles (see section 3 below) adds to the growing powers that domestic courts now enjoy (and/or assert) to scrutinise the output of democratic politics.^11^ But the domestication of judicial review powers to scrutinise administrative and legislative decision-making under the UKIMA is not simply a continuation of existing patterns of constitutional change. Read in conjunction with the UKIMA’s transformation of the UK’s material constitution (our second conclusion, above), it points to the normalisation of something distinctive: the introduction of judicial review to secure the foundations of a particular, market-orientated vision of the UK’s contemporary political economy.

## 2. Legal Transplantation: A Conceptual Framework

Legal transplantation features prominently in comparative law scholarship. Most notably, in his provocative study on the reception of Roman law within European legal systems, Watson proclaimed that legal transplants were legally unproblematic and ‘socially easy’.^12^ That conclusion triggered heated debate among comparative scholars on the feasibility of recognising legal transplantation as a distinct epistemological phenomenon. Critics, including Legrand, objected that talk of the movement of legal norms between systems as legal transplants was mistaken.^13^ What moved, he argued, were simply decontextualised propositions. This was due, he maintained, to the intimate link between legal norms and the specific legal environments which produced them and in which they operated. Broadly speaking, most comparative scholars now reason within the two extremes presented by Watson and Legrand, acknowledging, to different degrees, the significance of environmental conditions within both the exporting and receiving legal systems.^14^

Comparative legal scholars have been highly successful in developing legal transplantation as a theoretical framework. Its origins, however, stretch much further back in the history of ideas.^15^ Significantly for present purposes, those roots also engage constitutional discourse, not just discussion among legal historians and, primarily, private lawyers. Montesquieu, for instance, made direct appeals to the significance of internal environmental conditions as potential obstacles to the movement of legal ideas between legal systems.^16^ Contrastingly, writing several decades later, Bentham ascribed little significance at all to internal environmental conditions when theorising constitutions as basic frameworks of government throughout the Old and New Worlds.^17^ Like Watson thereafter, Bentham defended the movement between legal systems of constitutional norms rooted in enlightenment principles of liberal justice as something that was fundamentally easy.

This article develops the concept of legal transplantation as a framework to explore the impact of the UKIMA on the UK constitution. It draws on the existing work of comparative law scholars, but does not allow that scholarship to determine the scope of enquiry. Like most comparative scholars today, this article accepts the feasibility of legal transplants; in other words, it recognises, as a matter of principle and empirical practice, that legal norms may (and regularly do) transit between legal systems. Empirically, this is also visible in relation to pre-existing reforms to the UK constitution. Examples include the transplantation of fundamental rights norms from the ECHR into the UK legal system through the HRA.

The legal transplant framework developed in this article adds value at a conceptual level by focusing attention on a dual dynamic that structures the movement of ideas between legal systems, including, specifically, the transposition of EU internal market principles into the UK constitution through the UKIMA. Existing perspectives on the UKIMA (as well as other instances of legal transplantation, including the HRA) fail fully to capture that dynamic as a means to theorise and problematise patterns of domestic constitutional change. The focus, presently, is very much on rationalising constitutional innovations with established features of the UK constitution (notably the doctrine of parliamentary sovereignty) without regard to the conceptual framework on legal transplantation.^18^

On the one hand, the legal transplant framework directs legal scholars to assess the *impact of the UKIMA on pre-existing features of the UK constitution*. In other words, it provides a lens through which we may interrogate the impact of the EU internal market norms that the UKIMA transplants into the UK legal order on the nature of the UK constitution. Thus, engaging the conceptual framework on legal transplantation, we may reflect on the extent to which the UKIMA assumes the qualities of a constitutional Trojan Horse with the potential to leave a lasting imprint on the UK constitution. As section 3 will outline, the UKIMA’s design draws heavily on particular features of the EU internal market as the centrepiece of its highly constitutionalised supranational economic legal order. In so doing, that Act poses important challenges to established features of the UK constitution across its distinct territorial, material and conceptual registers.

On the other hand, this article engages the legal transplant framework to scrutinise the extent to which the UK constitution operates to reshape the basic characteristics of the EU internal market upon which the UKIMA unmistakably draws much of its substance. This enquiry goes far beyond an assessment of surface-level changes; for instance, differences with respect to the scope of application of the EU principles of mutual recognition and non-discrimination (see further section 3 below). The legal transplant framework cuts much deeper. It requires an assessment of the *impact of the receiving constitutional environment on the object of legal transplantation*. To what extent does the UKIMA as a legal transplant transform established EU internal market principles to align with existing features of the UK constitution? As section 4 will demonstrate, the unitary nature of the UK constitution coupled with the dynamics of executive dominance at Westminster has exercised a determinative effect on both the adoption and design of the UKIMA.

## 3. The United Kingdom Internal Market Act 2020

### A. The UKIMA: Aims and Structure

The UKIMA has four main objectives: first, to make provision for an internal market for goods and services within the UK, including in relation to the recognition of qualifications; secondly, to address the specific position of Northern Ireland post-Brexit; thirdly, to authorise the provision of financial assistance by the UK government to support, among other things, economic development and infrastructure projects throughout the UK; and finally, to reserve to the UK government exclusive competence to regulate the provision of state aid within the UK post-Brexit. The UK government maintained that legislation was necessary to address each of these objectives as a means to secure frictionless trade across the four nations of the UK following the UK’s exit from the EU internal market.^19^ In relation to Northern Ireland, the UKIMA served an additional important function in the implementation of the Protocol on Northern Ireland annexed to the EU/UK Withdrawal Agreement.^20^

Parts 1 and 2 of the UKIMA address goods and services. The overarching aim of both parts is to guarantee the free movement of these production factors across the four nations of the UK post-Brexit. The Act does this by mandating the prospective application of two fundamental principles—mutual recognition and non-discrimination—to all commercial transactions that fall within its scope. For example, with regard to mutual recognition, section 2 outlines that goods lawfully produced in, or imported into, one part of the UK where they may also be lawfully sold should, in principle, be able to be lawfully sold in all other nations of the UK. Statutory provisions that impose ‘relevant requirements’ that speak, among other things, to the particular characteristics of those goods or, likewise, to their production, presentation or packaging are prohibited. Parallel frameworks govern the application of the principle of non-discrimination in relation to goods and, by analogy, the application of mutual recognition and non-discrimination to the provision of services falling within the scope of the UKIMA.^21^

Part 3 establishes a unified system for the UK-wide recognition of professional qualifications. In parallel to the provisions on goods and services, this centres on the prospective application of two market access principles: automatic recognition (a variant of mutual recognition) and non-discrimination. Part 4 provides for the creation of a new Office for the Internal Market (OIM) within the existing Competition and Markets Authority. The OIM is charged with reporting responsibilities and is given powers to investigate the health of the UK internal market.^22^ It may also issue non-binding advice on the compatibility with the UKIMA of proposed regulations falling within that Act’s scope at the request of the UK government and/or the devolved administrations.^23^ Part 5 of the UKIMA addresses the particular place of Northern Ireland within the UK internal market in accordance with the terms of the EU/UK Withdrawal Agreement. During its passage through Parliament, this Part proved the most controversial, attracting strong criticism.^24^ Finally, Part 6 of the UKIMA establishes a new UK-wide framework for the provision of financial assistance to further economic development, improve infrastructure and support cultural, sporting and educational activities, including exchanges.

### B. The UKIMA as a Legal Transplant

The design and structure of the UKIMA borrows from its EU counterpart at a number of distinct levels. First, at a conceptual level, the UKIMA transplants into the UK legal order the basic idea of an internal market for goods and services. The UK government was right to assert in a White Paper that, prior to the UK’s exit from the Union, the UK already had a functioning internal market as a matter of domestic law.^25^ The Acts of Union, in particular, established the UK as a unified customs territory with a single currency.^26^ However, the pre-existing domestic internal market in no way reflected the basic design of the legal framework for continued economic integration that the UKIMA establishes. For one thing, during the UK’s period of EU membership, the UK legal system provided no domestic guarantees with respect to intra-UK market access for goods and services.^27^

Secondly, the nature of the UKIMA as an act of legal transplantation is apparent at the level of principle. As outlined above, that Act borrows the EU internal market principles of mutual recognition and non-discrimination to manage the movement of goods and services within the UK post-Brexit. Together with the unifying principle of ‘market access’, neither of these principles is, of course, peculiar to the EU legal order. Indeed, the language and logic of market access, mutual recognition and non-discrimination as substantive checks on cross-border trade now underpins most regional and international trade agreements.^28^ But a closer reading of the UKIMA evidences the proximity of the new domestic variants of these principles to their pre-existing EU counterparts. For example, to define the prohibited category of ‘relevant requirements’ regulating market access for goods, the UKIMA directly transposes the ECJ’s jurisprudence on mutual recognition.^29^

The UKIMA even goes so far as to transpose the *Keck* exception that the Luxembourg Court has formulated (and subsequently further qualified) in an attempt to manage the outer limits of article 34 TFEU.^30^ Following the French and German translations of that decision (‘Verkaufsmodalitäten’ and ‘modalités de vente’, respectively), section 3(4) UKIMA outlines that statutory measures regulating ‘manner of sale requirements’ are not prohibited *unless* they constitute disguised restrictions on intra-UK trade (transplanting the *Keck* exception)^31^ or impose unusually restrictive conditions that make it practically impossible to sell the relevant goods (transplanting the post-*Keck* refinement in *Motorcycle Trailers*).^32^

Further links to the structures and substantive principles of the EU internal market can be found throughout the UKIMA. Prominent examples include the Act’s reproduction of the rules on the provision of services and the recognition of professional qualifications in Directive 2006/123 EC.^33^ The UKIMA’s exclusions on the new domestic scope of those rules also continue to mirror the pre-existing EU legal framework. Similarly, schedule 2 of that Act draws on key instruments of secondary Union law, including the REACH Regulation (governing the production and use of chemical substances), to manage the scope of the mutual recognition principle in specific policy areas.^34^

As an act of legal transplantation, the UKIMA’s borrowing from the EU internal market model is not based on direct replication. For one thing, unlike its EU counterpart, the UK’s domestic internal market is limited to regulating the movement of goods and services, the recognition of certain professional qualification and aspects of subsidy control. By contrast, the EU internal market extends to guarantee the free movement of capital (art 63(1) TFEU) and persons (arts 45 TFEU), as well as the freedom of establishment (art 49 TFEU). Equally notable is the absence in the UKIMA of certain broader constitutional principles that animate the EU internal market.^35^ One prominent example is the subsidiarity principle (art 5(3) TEU).

We may approach discussion of distinctions between the design of the EU and UK internal markets simply as an exercise in comparative textual analysis. Likewise, we may appeal to considerations of political preference—in this instance, differences between the UK and EU internal markets may be theorised with reference to the objectives of a particular domestic majority Conservative government. The legal transplant framework, however, pushes legal scholars to take things much further. It directs us to do more than analyse textual differences and expressions of political preference. Rather, as the remainder of this article will demonstrate, the focus shifts to exploring the interplay of two powerful dynamics. This enquiry begins, in the next section, with an analysis of these dynamics in relation to the UK’s territorial constitution. To what extent is the UKIMA, as an act of legal transplantation, *shaping* and, conversely, *being shaped by* the nature of the UK’s distinctive territorial constitution?

## 4. Legal Transplantation: The UKIMA and the Territorial Constitution

We are accustomed to theorising the relationship between the UK and devolved administrations in terms of a division of legislative, administrative and executive functions within a unitary state.^36^ Under the Devolution Acts, the UK Parliament has transferred general powers to the devolved administrations, subject to a framework of reservations and limitations. Formally, the UK Parliament retains its competence to legislate in areas of devolved responsibility and, likewise, to adjust or even repeal the Devolution Acts in accordance with the doctrine of parliamentary sovereignty.^37^ As a matter of political practice, however, the UK government is committed to respecting the powers of the devolved administrations to act within their respective fields of competence and, accordingly, does not (or at least did not pre-Brexit) routinely intervene without the consent of the affected administration(s).^38^

### A. The Impact of the UKIMA on the UK Constitution as a Legal Transplant

With respect, first, to its impact *on the UK constitution*, the UKIMA introduces something genuinely novel into the UK’s pre-existing territorial constitution. It establishes the UK internal market as a shared regulatory space that *cuts across* the boundaries that the Devolution Acts set for the exercise of legislative, administrative and executive competences across the four nations of the UK. This is very different to the established mode of managing the UK’s territorial constitution and evidences the first of the two dynamics of legal transplantation in operation: EU internal market principles *shaping* the UK constitution.

In a departure from the familiar ‘devolve and forget’ approach to the distribution of administrative and legislative competences throughout the UK, the UKIMA is premised on a fusion of government powers, expressly unifying the UK and devolved administrations in pursuit of a common regulatory objective—the management of a UK-wide internal market. Substantively, this mirrors the logic of the EU’s internal market, the purpose of which is to unite the national markets of the 27 Member States into a single European market. If we were to assign this structure an overarching conceptual framework, it would be federal.^39^ The logic of both the EU and UK internal markets reflects the core of the federal idea: an attempt to balance self-rule with shared rule within a system of multilevel governance.

In its EU context, this federal dynamic involves balancing the regulatory autonomy of the Member States with the demands of the Treaty provisions on intra-EU movement. In the first instance, EU Member States are required to comply with the market access principles when exercising their legislative and administrative competences to regulate their respective national markets. The European Commission, Member States and individuals may invoke the Treaty provisions on intra-EU movement (specifically: art 34 TFEU on goods; art 45 TFEU on workers; art 49 TFEU on establishment; art 56 TFEU on services; and art 63(1) TFEU on capital) to challenge the compatibility of national measures with the principles of mutual recognition and non-discrimination.^40^ The UKIMA adopts the same basic approach to supervise the scope for regulatory divergence across the four nations of the UK, albeit requiring compliance with the market access principles only prospectively and in relation to the regulation of goods and services.^41^

At the EU level, the requirement imposed on Member States to comply with the demands of the Treaty provisions on intra-EU movement is complemented by the existence of a framework for the adoption of common rules that apply *throughout* the Union’s internal market. To borrow the jargon used to theorise the dual regulatory and de-regulatory dynamics that govern the EU internal market, negative integration (the requirement to comply with the market access principles) is accompanied by *positive integration* (the joint adoption of common EU standards).^42^ Article 114 TFEU establishes the primary procedure for the adoption of EU legislation to harmonise regulatory conditions throughout the EU internal market. That provision empowers the European Parliament and the Council of the European Union, acting on a proposal from the European Commission, to ‘adopt … measures for the approximation of the provisions laid down by law, regulation or administrative action in Member States which have as their object the establishment and functioning of the internal market’.

Strikingly, the UKIMA does not establish any comparable mechanism for positive integration; in other words, it does not provide a framework for the joint adoption, at UK-level, of harmonised provisions in relation to the movement of goods and services within the UK internal market. Whereas art 114 TFEU creates a framework for the adoption of common rules that apply *throughout* the Union’s internal market, the UKIMA only supervises the enactment of legislative and administrative measures by the UK Parliament and devolved administrations within their respective areas of competence (negative integration).

As Armstrong argues, by excluding positive integration, the UKIMA embodies a clear political choice to prioritise regulatory competition over intergovernmental cooperation in relation to the management of intra-UK trade in goods and services.^43^ The Act’s market access principles regulate the UK internal market as a shared regulatory space by forcing the UK Parliament (legislating for England) and the devolved administrations into potential competition with one another. Owing to the disproportionate size of its domestic market, this effectively means acknowledging the potential for English regulatory *dominance*. Contrastingly, the EU’s synthesis of negative and positive integration represents an attempt to balance the benefits of regulatory competition between the Member States with the application of jointly adopted EU instruments that establish common (typically minimum) standards to facilitate the free circulation of products and services throughout the Union.

Importantly, the framework for positive harmonisation is not entirely absent from the overall design of the UK’s new internal market. It just exists *outside* the UKIMA in the form of the Common Frameworks mechanism established in October 2017 by agreement of the UK government and devolved administrations.^44^ That agreement provides for the adoption of common approaches to the regulation of key policy areas that were previously governed at EU level and are now within areas of devolved competence.^45^ Under the 2017 Communiqué, common frameworks in these and other areas (154 in total)^46^ may consist of ‘common goals, minimum or maximum standards, harmonisation, limits on action, or mutual recognition, depending on the policy area and the objectives being pursued’.

The UKIMA governs the relationship between negative integration under the market access principles and positive integration under the Common Frameworks. That Act empowers the UK government to amend the UKIMA’s schedules to give effect to any jointly adopted Common Frameworks.^47^ Where that power is exercised, its effect is to suspend the application of the market access principles in specific policy areas to reflect the existence of intergovernmental consensus on common standards. In effect, hard law (the UKIMA) trumps soft law (the Common Frameworks). The next section (section 4B) will further discuss the implications of entrusting that important power to the UK government through the lens of legal transplantation.^48^

### B. The Impact of the UK Constitution on the UKIMA as a Legal Transplant

The design of UKIMA illuminates the impact of the EU internal market on the UK’s territorial constitution. In particular, it points to the introduction, by legal transplantation, of an inherently federal approach to managing the coexistence of different sites of legislative and administrative authority under devolution.^49^ But, as outlined in section 2 above, legal transplantation does not simply provide a framework to theorise the impact of EU internal market principles on the UK’s territorial constitution. It also focuses attention on a parallel dynamic: *the impact of the UK constitution on the UKIMA* as an act of legal transplantation. Accordingly, we must also ask: to what extent has the transplantation of EU internal market norms been conditioned by established principles of the UK constitution?

Two important characteristics of the UK’s territorial constitution have conditioned the reception of EU internal market principles and structures into the UK legal order through the UKIMA. The first is the UK constitution’s unitary nature. The establishment of the devolved administrations has created important new sites of legislative and administrative authority (as well as democratic accountability) within the UK.^50^ However, the pivot to pluralism through devolution has not been at the expense of the continued recognition of the legislative supremacy of the UK Parliament—both formally and, as the Brexit process has demonstrated, in substance. The UK Parliament retains its legislative supremacy and, conventions notwithstanding, is able to legislate in areas affecting the legislative and administrative competences of the Scottish Parliament, Welsh Senedd and Northern Ireland Assembly and their respective governments.^51^ The second defining characteristic is the UK government’s effective control of Parliament’s legislative supremacy.^52^ A strong majority in the House of Commons does not guarantee the smooth passage of primary legislation, but it empowers incumbent governments to control the legislative programme and, in effect, instrumentalise Parliament’s legislative supremacy to achieve their desired policy objectives.^53^

The unitary character of the UK constitution combined with the dynamics of majority rule at Westminster exercised a profound effect on the UKIMA as a legal transplant. It was this receiving environment that empowered and restrained the government’s political choices in relation to the establishment of a new domestic internal market. Its impact is visible at two different levels. First, it conditioned the *adoption* of the UKIMA. Secondly, it impacted directly on the *design* of that Act; specifically, its approach to configuring new frameworks for intergovernmental cooperation to manage the application of the market access principles across the four nations of the UK.

In relation to the adoption of the UKIMA, the unitary character of the UK constitution combined with the dynamics of majority rule at Westminster to lock the devolved administrations into the UK government’s approach to the domestication of EU internal market principles post-Brexit. Both the Scottish and Welsh governments fiercely opposed the UK government’s plans from the outset, arguing that these not only undermined devolution, but were also unnecessary given agreement reached in 2017 on the Common Frameworks and risked a lowering of regulatory standards.^54^ Their respective views were voiced publicly in the strongest of terms.^55^ Yet, under the UK constitution, the UKIMA could be lawfully enacted notwithstanding the refusal of the Scottish Parliament and the Welsh Senedd to pass consent motions approving the UK government’s Internal Market Bill. The co-option, by force of constitutional principle, of the devolved administrations into the UK internal market contrasts with the procedures governing the establishment of the EU internal market. In the latter case, the Member States freely consented to the adoption of the EU Treaties (and their subsequent amendment) as institutional partners. They were also broadly aligned politically in relation to the basic principles and structures of the EU internal market.

With respect to matters of design, the UK’s territorial constitution exercised a decisive impact on the UKIMA’s approach to structuring the management of the market access principles centrally; in other words, at UK level. A striking feature of the UKIMA’s approach to centralised market-management is its failure to recognise (and distinguish between) the duality of the UK government’s institutional roles vertically as both sub-national (English) and national (UK) regulator. This contrasts with the EU’s internal market structures, which maintain neat divisions between the institutional roles of Member State governments at both the national and Union levels, notably with respect to the adoption of common rules that apply throughout the Union’s internal market.^56^

The failure to differentiate between the duality of the UK government’s roles as sub-national and national regulator is not a new phenomenon.^57^ It reflects a familiar tension in the UK’s asymmetrical territorial constitution regarding the political representation of England as the largest of the four component nations.^58^ However, under the UKIMA, existing constitutional tensions assume far greater significance. In particular, the absence of clear regard for the UK government’s dual contribution as regulator at the sub-national (England) and national (UK) levels collides with the underlying federal logic of the internal market as a shared regulatory space that cuts across the boundaries that the Devolution Acts set for the exercise of legislative, administrative and executive competences across the four nations of the UK (see section 4A above). The effective functioning of that newly constituted market is premised on capturing that duality in institutional form.

The impact of the UK’s territorial constitution on the UKIMA as a legal transplant is further apparent horizontally; in other words, with respect to relations between the UK government and the devolved administrations. Impressed throughout the UKIMA is the primacy of the UK government as UK-wide regulator. First, in relation to negative integration, the UK government retains a significant degree of control over the scope of the mutual recognition and non-discrimination tests.^59^ This is evident, for example, through its powers to amend the definition of ‘relevant requirements’ for goods, as well as to modify the frameworks governing the justification of measures that infringe either principle.^60^ Prior to exercising those powers, the Secretary of State is required to seek the consent of the devolved administrations, but may proceed regardless after one month provided she provides a reasoned statement.^61^ Secondly, with respect to the adoption of UK-wide rules in areas of concurrent competences (positive integration), the UKIMA grants the UK government an important power to adjudicate on the application of any rules that are jointly agreed by the UK government and devolved administrations through the Common Frameworks mechanism. As foregrounded above (section 4A), section 10(3) UKIMA empowers the UK government ultimately to determine the extent to which any Common Framework addressing the regulation of goods and services should be exempted from the application of the market access principles under that Act.^62^

The priority the UKIMA affords to the UK government as central (UK) regulator in relation to both negative and positive integration draws some legitimacy from the existence of UK-wide elections to the Westminster Parliament.^63^ But it would also appear to conflate Parliament’s legislative supremacy (classically viewed as indivisible) with *executive supremacy* at Westminster. The UK Parliament does indeed retain, at least formally, its supremacy to legislate for the whole or part of the UK, including in areas of devolved competences affecting the UK internal market. But it does not follow from this that *the UK government* ought to retain ultimate control over the regulation of that market.^64^ As the Scottish government has outlined, ‘devolution is not about a hierarchy of Governments; it is about a hierarchy of Parliaments’.^65^

There is, of course, nothing new in recognising deficiencies in the design of frameworks for joined or shared government between the UK government and the devolved administrations.^66^ This remains a defining feature of the UK’s asymmetrical territorial constitution—the receiving constitutional environment that shaped the reception of the UKIMA into the UK legal order as a legal transplant. It reflects a particular legal understanding of the unitary constitution under the doctrine of parliamentary sovereignty, the legitimation of that doctrine through UK-wide elections to the UK Parliament and the size of the English nation.^67^ It is also, in part, the result of a broader historical legacy that has tended to prioritise the maintenance of difference within a ‘union state’ over the establishment of robust (and, in particular, federal)^68^ frameworks for joint and shared government.^69^

Nonetheless, one may recognise this broader constitutional context yet still express dissatisfaction with the adoption and design of the UKIMA. Even if it aligns with existing constitutional principles, the regulatory framework that Act establishes by legal transplantation exacerbates rather than challenges existing asymmetries in the UK’s territorial constitution. If the prospect of establishing a UK internal market post-Brexit presented fresh opportunities for innovation in relation to the UK’s territorial constitution, the UKIMA represents a rather disappointing half-baked response.

## 5. Legal Transplantation: The UKIMA and the Material Constitution

This section engages the legal transplant framework to assess the impact of the UKIMA on the UK’s material constitution. To a greater degree than in the previous section (which assessed the territorial constitution), it accentuates the susceptibility (or, put another way, ‘openness’) of the UK constitution to potentially far-reaching change by legal transplantation. In summary, the UKIMA introduces into the UK constitution a powerful commitment to a market economy structured around the market access principles. That commitment, which it borrows from the EU legal order, is previously absent from the UK’s domestic constitutional arrangements. Its effect on legislative politics across the UK is uneven. Whereas the devolved legislatures must comply with the demands of the UKIMA, the UK Parliament (regulating the English market) remains ultimately and uniquely free to sidestep the market access principles on a case-by-case basis in accordance with the doctrine of parliamentary sovereignty.

### A. Variants of Power: The Material Constitution

The material constitution speaks to considerations of substantive functioning.^70^ It focuses on the objectives that constitutions are designed to achieve as basic frameworks of government. Theorising substantive goals, constitutional scholars typically distinguish principally between the performance of negative and positive functions.^71^

Classically, constitutions are associated, negatively, with restraints on government power. In the dominant liberal tradition, constitutions appear as instruments that exist to constitute the state and the institutions of government; legitimate the exercise of government power (typically through elections with universal suffrage); and, finally, protect basic civil and political rights as a means to enable citizens to pursue their particular conceptions of the good life whilst maximising the freedom of others to do the same.^72^ Beyond these basic functions, scholars also recognise the potential for constitutions to function, positively, as instruments designed to facilitate the realisation of specific economic, political and social objectives. Thus, rather than simply protect individuals from excesses of government power, constitutions can and, for many, should also commit the exercise of that power to the achievement of particular substantive ends.^73^

The UK constitution speaks principally to the negative pole in terms of its substantive functions. As such, it remains focused on guaranteeing the protection of basic civil and political liberties and, more recently, fundamental rights. Historically, these guarantees emerged in the case law of the courts, which directed the common law towards the protection of individual liberty through restraints on government power.^74^ The Human Rights Act—incidentally, another legal transplant—effected an important change in approach in relation to the protection of civil and political liberties. That Act transformed the protection that the UK constitution affords citizens in that sphere from negative safeguards into positive constitutional rights.

Beyond the protection of basic civil and political liberties and fundamental rights under both the HRA and the common law, however, the UK constitution is notably silent in terms of its pursuit of specific substantive objectives. Indeed, it remains a distinguishing feature of the UK constitution under the doctrine of parliamentary sovereignty that majority governments enjoy considerable freedom to effect potentially far-reaching political, economic and social change without constitutional limitation.^75^ Provided their objectives do not conflict with the constitutional protections set out in the HRA or recognised in the common law, successive majority governments may, for example, oscillate violently between the pursuit of competing reform programmes with transformative effects on the UK’s political economy.^76^ For some, this remains a source of considerable strength,^77^ which, historically, has also been exploited—with varying degrees of success—by both socialist and conservative governments alike.

The European Union’s material constitution, which structures the operation of its internal market, reflects a very different variant of power that permits no such degree of normative flexibility. The exercise of governmental power at Union level is firmly anchored to the realisation of a clearly articulated set of substantive constitutional objectives designed to secure integration between the Member States.^78^

The EU treaties commit the Union institutions and the Member States to a programme of deep economic and political integration. The original EEC Treaty established the foundations of an economic constitution that is based on a legal ordering of the economies of the Member States in accordance with the principles of free movement and undistorted competition. The EU internal market (formatively the Common Market) remains the centrepiece of that constitution. Originally intended to be achieved through a programme of legislative initiatives controlled by political actors (including representatives of Member State governments), the Common Market project was quickly captured by the ECJ as part of its broader effort to transform the founding EEC Treaty into a ‘new legal order’.^79^ Indeed, it was the EEC Treaty’s provisions on intra-EU movement that provided the Luxembourg Court with the oxygen it required to constitutionalise the EU legal order by asserting the direct effect and primacy of Union law.^80^

In substantive terms, the EU’s economic constitution places significant limits on the exercise of public power at both Member State and Union levels. It also restrains private actors to the extent that their conduct triggers the application of the EU’s competition law framework or, to a lesser extent, is judged to fall within the scope of the Treaty provisions on intra-EU movement.^81^ In relation to the latter, the ECJ interprets the market access principles—mutual recognition and non-discrimination—remarkably broadly to capture (typically Member State) measures that, in principle, often need do no more than simply make intra-EU trade ‘more difficult’ or ‘less attractive’.^82^ Such measures may be justified with reference to an open-ended list of non-economic overriding public interest requirements. But justifications are subject to a strict proportionality test that, structurally, operates to privilege market objectives.^83^

### B. The UKIMA: Reshaping the Economic Constitution

The UKIMA transplants the market access principles into the UK constitution as enforceable limits on the exercise of administrative and legislative power. In so doing, that Act introduces a powerful new commitment to the principles of a liberal market economy as the basis of the UK’s material constitution. That commitment is qualitatively different to earlier reforms; for example, the reconceptualisation of civil liberties as fundamental rights in both the common law and under the HRA. It is also qualitatively different to the application of the market access principles domestically throughout the period of the UK’s membership of the EU internal market.

In comparison to the UKIMA, the European Communities Act 1972 never fully integrated the EU’s economic constitution into the UK’s own material constitution. The scope of application of the EU’s market access principles (and accompanying rules on EU competition policy) applied only to intra-EU trade (and intra-EU anti-competitive practices), leaving the regulation of trade in goods and services between the four nations of the UK a matter for domestic law. Accordingly, it was, for example, entirely lawful for the Scottish government to introduce legislation on minimum alcohol pricing provided this complied with article 34 TFEU on the free movement of goods.^84^ As a matter of domestic law, compliance with EU law (and the Devolution Acts) was all that mattered.

On the one hand, the priority that the UKIMA now affords to the protection of the market economy over competing non-market preferences is narrower in scope than the position under the EU Treaties: the UKIMA regulates only goods and services. Its principles also apply only prospectively.^85^ On the other hand, however, the underlying commitment to the market economy is much stronger. The UKIMA permits a much narrower set of derogations to the market access principles than those applying at the EU level. The principal derogations it recognises are restricted to considerations of human, animal or plant health along with a range of specific exceptions. In substantive terms, this contrasts with the EU internal market model, which recognises, at least in principle, the potential to justify restrictions on the free movement of goods and services with reference to an extremely broad and non-exhaustive list of proportionate public interest requirements (‘mandatory requirements’), including, for example, environmental and consumer protection, maintaining national and regional diversity, and fundamental rights.^86^ Institutionally, it also reflects a significant change in approach. At EU level, it is the ECJ that adjudicates on the availability and application of derogations to the market access principles on a case-by-case basis. Under the UKIMA, the UK government retains control of the justification framework.^87^

Recognising the UKIMA’s impact on the UK’s material constitution has important consequences for legislative politics. In common with the EU experience, the domestication of the market access principles functions to restrain the scope for democratic majoritarian politics in line with liberal economic principles.^88^ This is first and foremost a challenge for the devolved administrations. Indeed, owing to the doctrine of parliamentary sovereignty, the UK Parliament (regulating the English market) ultimately retains its competence to legislate contrary to the market access principles; for example, through the use of ‘notwithstanding’ clauses. In relation to the devolved legislatures specifically, the UKIMA also amends the Devolution Acts, adding that statute to the list of ‘protected’ instruments that the devolved legislatures are precluded from modifying. Following the approach under the EU internal market, conflicts between sub-national regulations and the market access principles do not render the former unlawful (*ultra vires*).^89^ Rather, under sections 2(3) and 5(3) of the Act, the relevant measures are simply ‘disapplied’ to the extent that they apply to goods and services lawfully circulating or provided elsewhere within the UK market.

The UKIMA’s domestication of key features of the EU’s economic constitution in the above manner creates space for the replication of the institutional conflicts that play out between Member States in relation to the management of the Union’s internal market. In parallel with Member State governments, the Scottish, Welsh and Northern Irish administrations, in particular, are now forced to justify policy preferences that interfere with the market access principles. As outlined above, unlike the UK Parliament (regulating the English market), the devolved legislatures are unable expressly to override those principles; for example, through the use of notwithstanding clauses. To the extent that their respective policy preferences may not be justified under the UKIMA, the devolved legislatures may therefore ultimately be bounced into deciding whether or not to apply their proposed regulations purely internally; in other words, within their particular sub-national market.^90^

That the devolved administrations have reacted so critically to the UKIMA should not surprise.^91^ That Act’s modifications to the UK’s material constitution have significant implications for the exercise of their respective competences. The market access principles may not preclude the devolved administrations from legislating in the same way that, for example, the Devolution Acts make it unlawful (*ultra vires*) for the Scottish Parliament, Welsh Senedd and Northern Ireland Assembly to enact legislation this is contrary to Convention rights. Nonetheless, their prospective application under the UKIMA imposes significant practical limits on their political autonomy in areas of devolved competence—limits that the dominance of the far larger English market further reinforce. In substantive terms, the market access principles are also far more onerous than other positive obligations governing the exercise of public power such as the Public Sector Equality Duty that, on one view, arguably also now form part of the UK’s evolving material constitution.^92^

What is more, as section 4 concluded, the UKIMA does not compensate for this encroachment into sub-national regulatory autonomy by establishing effective structures for shared or joint government that provide space for genuine UK-wide agreement on the appropriate limits to the new domestic market paradigm. The only mechanism for consensual intergovernmental cooperation—the Common Frameworks—exists outside the UKIMA as a soft law instrument and is, ultimately, subordinate to the UK government’s powers under that Act.

## 6. Legal Transplantation: The UKIMA and the Conceptual Constitution

This section turns, finally, to consider the implications of the UKIMA for the UK’s conceptual constitution. It reaches two conclusions. First, it argues that the UKIMA effects a qualitative change to established patterns of judicial review through its co-opting of courts as agents to secure the foundations of the newly recast material constitution. Secondly, in broader terms, this section reveals a striking lack of political contestation at Westminster surrounding the creation of this significant new judicial role. This suggests a high degree of political consensus at Westminster in relation to the fundamentals of the UK’s contemporary political economy.

### A. The UKIMA and the Conceptual Constitution

Traditionally theorised as political in character,^93^ the UK constitution now incorporates a range of features that are classically associated with opposing models of constitutional design. Some of the features, notably the judicial review of legislation, are the result of Parliament’s institutional choices. As such, one may arguably defend these as expressions of the political constitution in operation. Examples include the establishment of judicial review powers under the HRA and Devolution Acts, respectively.^94^ Other developments, however, pose stronger challenges to the orthodox view of the UK constitution under the doctrine of parliamentary sovereignty. Chief amongst these innovations is the emerging body of jurisprudence gesturing towards the judicial review of primary legislation with reference to the principle of legality; in other words, judicial review in opposition to (rather than in furtherance of) Parliament’s statutory instructions.^95^

As a legal transplant, the UKIMA represents the continuation of the former trend: the judicialisation of democratic politics by legislative instruction (Act of Parliament). The UKIMA provides a new domestic basis for courts to engage in the scrutiny of administrative and legislative decision-making for compliance with the market access principles. Specifically, its attribution of direct effect to those principles (see eg sections 2(3) and 5(3)) opens up the possibility for private litigants to secure the disapplication of legislative measures that interfere with intra-UK trade in goods and services.^96^ This model of private enforcement mirrors the EU approach to managing regulatory divergence within the EU internal market in accordance with the case law of the ECJ (section 4A above).

On the one hand, there is nothing particularly striking about the establishment of new judicial review powers, including in relation to Acts of the UK Parliament. These powers are well-established attributes of the UK constitution. As noted, the Devolution Acts, for example, empower courts to scrutinise the legality of Acts of the devolved legislatures for compliance with the limits on their competences set out therein. Prior to the UK’s exit from the EU, these limits included a requirement to comply with EU law.^97^ Likewise, the HRA provides a softer framework to legitimate the judicial review of Acts of the UK Parliament whilst respecting the doctrine of parliamentary sovereignty. Before its repeal, the European Communities Act 1972 also empowered domestic courts to review Acts of both the UK Parliament and the devolved legislatures for compliance with EU norms—with domestic courts acting as ‘community courts’ to ensure compliance with Union law.^98^

On the other hand, however, the UKIMA does not simply represent the continuation of embedded constitutional patterns in relation to judicial review. That Act introduces something new into the UK’s evolving conceptual constitution. It extends judicial control over administrative and legislative decision-making into a new area: the defence of the UK’s material constitution; specifically, the protection of the liberal market economy (section 5 above). In short, the UKIMA tasks courts with the responsibility to ensure the application of the market access principles as cornerstones of the UK’s post-Brexit material constitution.

Substantively, the instruction given to the courts reflects a purer vision of the political market economy than that animating the EU’s internal market. In comparison with the ECJ’s jurisprudence, the UKIMA affords UK courts far less discretion to balance the protection of intra-UK trade with non-economic values in instances of conflict.^99^ As already outlined (section 4B above), the UKIMA does not provide for an open-ended and judicially managed category of proportionate ‘mandatory requirements’ to balance the demands of intra-UK movement with competing non-economic considerations.^100^ Added to this, the UK government has also largely reserved to itself the task of making any future adjustments to the scope of the market access principles (section 4B above).^101^

### B. The UKIMA and Constitutional Politics

The relationship between the UKIMA and the UK’s conceptual constitution extends much further than the identification of qualitative changes to existing domestic judicial review frameworks. In broader terms, that Act also offers important insights into the fault lines that delineate contemporary constitutional politics at Westminster. A striking feature of the UKIMA’s passage through Parliament is the extraordinary silence that accompanied the introduction of that Act’s provisions on judicial review. In contrast to the position under the HRA, parliamentarians (and legal scholars) attached comparatively little significance to the UKIMA’s domestication of the market access principles as directly effective norms against which the constitutionality of administrative and legislative measures may be scrutinised before the courts. Indeed, only one notoriously Eurosceptic MP railed forcefully against the domestication of judicial review powers post-Brexit.^102^ With respect to the UKIMA specifically, the political and scholarly spotlight was firmly on discussing the draft legislative provisions addressing Northern Ireland. That was the hot topic.

We may attach varying degrees of significance to parliamentarians’ apparent comfort with the domestication, through the UKIMA, of the judicial review powers that UK courts previously exercised in accordance with the case law of the ECJ. As a continuation of powers derived (from a domestic constitutional perspective) from section 2 of the European Communities Act 1972, the UKIMA provisions on the direct effect of the market access principles arguably represent nothing new. Contrastingly, their very preservation as a component part of the UK’s constitutional framework post-Brexit may also be read, at the level of constitutional politics, as something much more radical: as a statement on the normalisation of an institutional role for courts in the scrutiny of democratic decision-making. Where once the prospect of empowering courts to scrutinise democratic politics agitated many parliamentarians (and majority governments), the enactment of the UKIMA would appear to signal that the establishment of judicial review functions—including in relation to primary legislation^103^—is no longer considered a controversial exercise of Parliament’s legislative supremacy. A triumph for liberal constitutionalism.

The weakness of the latter interpretation, of course, is that it jars with broader constitutional developments. The Brexit process has intensified tensions between Parliament and the courts. Recall here, for example, factions within Parliament publicly criticising judicial decisions on the scope of prerogative powers in response to the UK Supreme Court’s decisions in *Miller* and *Miller/Cherry*.^104^ Predating this, Conservative politicians, in particular, have also long had the curtailment of judicial powers in their sights; initially, in relation to the application of the HRA, and more recently (once again) with reference to the common law framework on judicial review.^105^ Strands of legal scholarship have also aligned with these critical perspectives at various junctures.^106^

An alternative interpretation appeals to deeper substantive considerations to square the circle. It reads parliamentarians’ relative silence on the domestication of judicial review powers under the UKIMA as an expression of an underlying political consensus regarding the fundamental nature of the UK’s contemporary political economy. The fact that the UKIMA’s domestication of judicial review powers to defend liberal market principles did not agitate politicians across the political spectrum points to the existence of broad cross-party support for the basic tenets of the market-orientated political economy that the Act transplants from the EU into the UK constitution (section 5 above). If that view is indeed correct, it invites us to consider the UKIMA’s establishment of new judicial review powers in relation to the market access principles as more than simply a continuation of existing patterns of constitutional change.

Read in conjunction with the UKIMA’s transformation of the UK’s material constitution, it points to the normalisation of something new and distinctive: the introduction of judicial review to secure the foundations of a particular, market-orientated vision of the UK’s contemporary political economy. On that reading, as directly enforceable norms, the market access principles assume the qualities of a new Parliament-endorsed baseline to circumscribe the legitimate scope of majoritarian legislative politics in accordance with liberal economic principles. As outlined above (section 5B), this is first and foremost a challenge for the devolved legislatures, which, unlike the UK Parliament, are unable to sidestep judicial applications of the market access principles in specific instances; for example, through the use of notwithstanding clauses.

Looking ahead, it remains to be seen just how significant the new judicial powers that the UKIMA establishes to uphold the market access principles will prove in practice. The OIM’s first report on the operation of the UK internal market cites no evidence of substantial policy divergence emerging between the four nations since 31 December 2020.^107^ But, as the OIM further outlines, that position is anticipated to change over time, with the potential for regulatory divergence across the UK internal market identified in six key areas: agriculture; animal welfare; energy use; environment; food and drink; and health and safety matters. Before both the Divisional Court and Court of Appeal, counsel for the Welsh government has already pointed to the potentially restrictive effects of the UKIMA on its competence to enact new legislation within these policy fields—specifically, to regulate single-use plastic.^108^

The extent to which the courts will be called upon to scrutinise new regulations against the market access principles will also depend on the effectiveness of soft law instruments. In particular, further meaningful progress towards the establishment of Common Frameworks may reduce pressure for recourse to the courts as a means to manage divergence within the UK internal market.^109^ Similarly, the OIM’s powers to prepare independent expert assessments on the economic impact of new regulatory measures on the UK internal market may assist with the resolution of intergovernmental disputes politically rather than judicially.^110^

Nonetheless, the (as yet) limited existence of regulatory divergence within the UK internal market, coupled with the availability of key soft law instruments to manage (and, in case of the OIM, support) joint decision-making, does not alter the underlying conceptual point advanced in this section. The overarching commitment to the basic tenants of a liberal political economy enshrined in the UKIMA performs a powerful signalling function, pointing to consensus at Westminster regarding the fundamentals of the UK’s contemporary political economy. It also co-opts courts into robustly defending that commitment throughout the UK internal market in an important agency role.

## 7. Conclusion

This article has developed the comparative law framework on legal transplantation to assess the impact of the UKIMA on the UK constitution across three distinct registers of constitutional analysis: the territorial, the material and the conceptual. The conclusions reached point to both the resilience of established features of the UK constitution and its susceptibility (or ‘openness’) to change by legal transplantation.

Resilience is particularly evident in relation to the UKIMA’s impact on the territorial constitution. Rather than replicate the EU’s model of federal market governance, the UKIMA has adopted an incomplete, coercive and structurally asymmetric approach to manage the UK internal market. Reflecting established trends and tensions in UK constitution law, the UKIMA is an instrument that centralises political authority, first and foremost in the hands of the UK government, which steered the Bill through Parliament. The existence—outside the framework of the UKIMA—of the Common Frameworks may go some way to resolve practical problems of intra-UK regulation post-Brexit. Those frameworks provide a mechanism for positive harmonisation that is largely absent in the UKIMA. They are also, by design, more consensual and collaborative. Nonetheless, what the Common Frameworks establish, the UKIMA takes away by empowering the UK government ultimately to rule on the application of the market access principles to any Frameworks establishing common standards by intergovernmental agreement.

As an act of legal transplantation, the UKIMA effects a profound impact on the nature of the UK’s material constitution. The Act introduces, for the first time, a strong commitment to the market economy that mirrors that underpinning the EU’s internal market. Its domestication of a powerful commitment to the market access principles reflects a particular vision of the political economy that is based on a substantively thicker version of the dominant liberal paradigm. The UKIMA expands the UK’s substantive constitution beyond the protection of civil and political liberties to defend a set of market access principles as enforceable ‘rights to trade’. The significance attached to that vision spills over to the conceptual constitution, with the UKIMA recognising a continued role for domestic courts to secure the basic tenants of the newly recast liberal market economy, albeit in modified form. For those uncomfortable with the further judicialisation of UK politics, this marks a worrying development. The UKIMA establishes a new framework to challenge majoritarian decision-making within and across the four nations of the UK. This is first and foremost a challenge for the devolved legislatures to the extent that they desire to adopt regulatory policies that conflict with considerations of economic efficiency.

But the legal transplantation framework does more than just expose sites of resilience, transformation and tension with respect to the UKIMA and the UK’s territorial, material and conceptual constitution. The framework’s dual dynamic directs legal scholars to scrutinise the potentially far-reaching and transformative constitutional effects associated with any attempt—consciously or unconsciously—to import legal norms into the UK legal system. As such, it is a powerful tool that is capable of generating further insights into the UK’s evolving constitutional arrangements and processes of domestic constitutional change.

## Footnotes

1 See eg European Union (Withdrawal) Act 2018; European Union (Withdrawal Agreement) Act 2020.

2 See eg United Kingdom Internal Market Act 2020; Fisheries Act 2020; Trade Act 2021.

3 European Union (Withdrawal) Act 2018, s 1.

4 On the new domestic source, see UK Withdrawal from the European Union (Legal Continuity) (Scotland) Bill Reference [2018] UKSC 64 [31].

5 On legal transplants, see eg Alan Watson, *Legal Transplants: An Approach to Comparative Law* (2nd edn, University of Georgia Press 1993); P Legrand, ‘The Impossibility of “Legal Transplants”’ (1997) 4 MJ 114; Sujit Choudhry (ed), *The Migration of Constitutional Ideas* (CUP 2007).

6 Case 26/62 *Van Gend en Loos* ECLI:EU:C:1963:1.

7 See also T Horsley, ‘The Migration of Constitutional Ideas Between and Beyond States: Adjectives, Analogies and Added Value’ in Anat Scolnicov (ed), *Constitutional Transplantations: The Diffusion and Adoption of Constitutional Ideas* (Hart Publishing forthcoming).

8 In relation to the domestication of ECHR rights through the Human Rights Act 1998, see eg Alison L Young, *Parliamentary Sovereignty and the Human Rights Act* (Hart Publishing 2009).

9 On trade as a unifying dynamic under the UK constitution prior to EU membership, see James Bryce, *Studies in History and Jurisprudence* (Clarendon Press 1901), 231.

10 See eg Case C-438/05 *International Transport Workers’ Federation v Viking Line ABP* ECLI:EU:C:2007:772, para 45; Case C-112/00 *Schmidberger* ECLI:EU:C:2003:333, para 74. For criticism, see eg M Hopner and SK Schmidt, ‘Can We Make the European Fundamental Freedoms Less Constraining? A Literature Review’ (2020) Cambridge Yearbook of European Legal Studies 182; Dieter Grimm, *The Constitution of European Democracy* (OUP 2017).

11 eg under the Human Rights Act 1998, ss 3 and 4; Scotland Act 1998, s 29.

12 Watson (n 5). See also Roscoe Pound, *The Formative Era of American Law* (Little Brown & Co Press 1938) 94: ‘the history of a system of law is largely a history of borrowing of legal materials from other legal systems and of assimilation of materials from outside the law’.

13 Legrand (n 5). See also O Kahn Freund, ‘On the Uses and Misuses of Comparative Law’ (1974) 37 MLR 1; JW Cairns, ‘Watson, Walton, and the History of Legal Transplants’ (2013) 41 Ga JInt’l & Comp L 637. See also recently Rosalind Dixon and David Landau*, Abusive Constitutional Borrowing: Legal Globalization and the Subversion of Liberal Democracy* (OUP 2021).

14 See eg V Perju, ‘Constitutional Transplants, Borrowing, and Migrations’ in Michel Rosenfeld and András Sajó (eds), *Oxford Handbook on Comparative Constitutional Law* (OUP 2012).

15 For a recent historical survey, see Linda Colley, *The Gun, the Ship, and the Pen: Warfare, Constitutions and the Making of the Modern World* (Profile Books 2021). See also discussion of the ‘domestic analogy’ in international law in eg *Hobbes’s Leviathan* (reprint, Clarendon Press 1965); Hidemi Suganami, *The Domestic Analogy and World Order Proposals* (CUP 1989).

16 Anne M Cohler, Basia C Miller and Harold S Stone (eds), *Montesquieu: The Spirit of the Laws, Cambridge Texts in the History of Political Thought* (CUP 1989) 8.

17 J Bentham, *Codification Proposal, Addressed by Jeremey Bentham to All Nations Professing Liberal Opinions* (London, 1822), cited in Philip Schofield and Jonathan Harris (eds), *The Collected Works of Jeremy Bentham: ‘Legislator of the World’: Writings on Codification, Law, and Education* (OUP 1998) 244.

18 See eg Young (n 8); Aileen Kavanagh, *Constitutional Review under the UK Human Rights Act* (CUP 2009).

19 See *UK Internal Market* (White Paper, CP 278, 2020).

20 Protocol on Ireland/Northern Ireland [2020] OJ L27/102, implemented by the European Union (Withdrawal Agreement) Act 2020.

21 See, respectively, UKIMA, ss 5–8 and ss 19–21. For analysis, see KA Armstrong, ‘The Governance of Economic Unionism after the United Kingdom Internal Market Act’ (2022) 85 MLR 635.

22 UKIMA, s 33.

23 ibid ss 34, 35 and 36.

24 In its original form, the draft Bill (cl 45) would have granted the UK government powers to suspend the operation of parts of the Northern Ireland Protocol—placing the UK in breach of international law. Elliot rightly described this as ‘constitutional dynamite’. M Elliot, ‘The Internal Market Bill—A Perfect Constitutional Storm’ (*Public Law for Everyone*, 9 September 2020) <https://publiclawforeveryone.com/2020/09/09/the-internal-market-bill-a-perfect-constitutional-storm/> (accessed 12 January 2022). See also eg House of Lords European Select Committee, *The United Kingdom Internal Market Bill: Part 5* (2020, HL 147).

25 Introducing the draft Bill to Parliament, see also Boris Johnson, HC Deb 14 September 2020, vol 680, col 41.

26 See Acts of Union 1707, arts VI, XVI and XVIII.

27 Intra-UK discrimination was also not precluded as a matter of Union law.

28 For discussion in relation to the WTO, see eg E Vranes, ‘The WTO and Regulatory Freedom: WTO Disciplines on Market Access, Non-discrimination and Domestic Regulation Relating to Trade in Goods and Services’ (2009) 12 Journal of International Economic Law 953.

29 UKIMA, s 3 lists as prohibited relevant requirements, statutory measures that relate, among other things, to the characteristics of the goods themselves or their production or presentation, as well as those imposing inspection, registration or approval requirements. This is lifted directly from the ECJ’s case law interpreting art 34 TFEU. See eg Case 120/78 *Cassis de Dijon* ECLI:EU:C:1979:42; see also Case 261/81 *Walter Rau v De Smedt PVBA* ECLI:EU:C:1982:382; Case C-170/07 *Commission v Poland* ECLI:EU:C:2008:322.

30 Joined Cases C-267/91 and C-268/91 *Keck and Mithouard* ECLI:EU:C:1993:905.

31 It is noteworthy that the UK supported the application of the *Keck* test and championed (unsuccessfully) its extension before the ECJ. See eg Case C-98/01 *Commission v United Kingdom (Golden Shares)* ECLI:EU:C:2003:273, para 45.

32 Case C-110/05 *Commission v Italy (Motorcycle Trailers)* ECLI:EU:C:2009:66.

33 Directive 2006/123 (EC) [2006] OJ L376/36.

34 Regulation 1907/2006 (EC) [2006] OJ L396/1.

35 Beyond this, the development of the UK internal market also took account of non-EU market models, including Canada. See eg *UK Internal Market* (n 19).

36 This remains the dominant approach to conceptualising the UK’s territorial constitution, at least among legal scholars. See eg M Elliot, ‘Legislative Supremacy in a Multidimensional Constitution’ in Mark Elliot and David Feldman (eds), *The Cambridge Companion to Public Law* (CUP 2015), 77. Contrast eg Baroness Hale, ‘The Supreme Court in the UK Constitution’ (Legal Wales Public Lecture, 12 October 2012) 23.

37 In relation to devolved competences, see Northern Ireland Act 1998, s 5(6); Scotland Act 1998, s 28(7); Government of Wales Act 2006, s 107(5).

38 The Sewel convention is not judicially enforceable, including in statutory form. See *R (on the application of Miller and another) v Secretary of State for Exiting the European Union* [2017] UKSC 5, [2017] 2 WLR 583 [151].

39 On federalism and devolution, see N Walker, ‘Beyond the Unitary Conception of the United Kingdom Constitution?’ [2000] PL 384; S Tierney, ‘Drifting Towards Federalism: Appraising the Constitution in Light of the Scotland Act 2016 and the Wales Act 2017’ in Robert Schütze and Stephen Tierney (eds), *The United Kingdom and the Federal Idea* (Hart Publishing 2018).

40 See, respectively, arts 258 and 259 TFEU, together with the case law of the European Court of Justice on the direct effect of the EU free movement provisions, eg *Van Gend en Loos* (n 6).

41 This is given effect in the substance of Parts 1 and 2 of the UKIMA (see section 3 above). On the direct effect of the UK market access principles, see section 5B below.

42 See eg Fritz Scharpf, *Governing in Europe: Effective and Democratic?* (OUP 1999) 43.

43 See Armstrong (n 21), who characterises this as the basis of a new model of ‘economic unionism’ within the UK.

44 See Joint Ministerial Committee (EU Negotiations) Communiqué, 16 October 2017 <https://assets.publishing.service.gov.uk/government/uploads/system/uploads/attachment_data/file/652285/Joint_Ministerial_Committee_communique.pdf> accessed 21 January 22.

45 Examples include public procurement; animal health and welfare; fisheries management and support; nutritional labelling, composition and standards; motor insurance; and food and feed safety and hygiene.

46 Frameworks Analysis 2020: Breakdown of areas of EU law that intersect with devolved competence in Scotland, Wales and Northern Ireland (Cabinet Office 2020) <https://assets.publishing.service.gov.uk/government/uploads/system/uploads/attachment_data/file/919729/Frameworks-Analysis-2020.pdf> accessed 21 January 22.

47 UKIMA, s 10(3), read in conjunction with s 10(4).

48 I am grateful to the anonymous reviewer for raising the soft/hard law distinction here.

49 See also S Tierney, ‘The Territorial Constitution and the Brexit Process’ (2019) 72 Current Legal Problems 59.

50 See Walker (n 39).

51 In addition to the UKIMA, the EU Withdrawal Act 2018, EU (Withdrawal Agreement) Act 2020 and European Union (Future Relationship) Act 2020 were all adopted without the consent of at least one of the devolved administrations as required under the Sewel Convention.

52 See, notably, Lord Hailsham’s characterisation of an ‘elective dictatorship’. Lord Hailsham, *The Dilemma of Democracy: Diagnosis and Prescription* (Collins 1978).

53 See T Endicott, ‘The Stubborn Stain Theory of Executive Power: From Magna Carter to *Miller*’ (Policy Exchange 2017); S Laws and R Ekins, ‘Endangering Constitutional Government’ (Policy Exchange 2019).

54 In relation to the Scottish government, see eg Constitution and Cabinet Directorate, ‘UK Internal Market: Initial Assessment by the Scottish Government (July 2020). For the Welsh perspective, see eg External Affairs and Additional Legislation Committee, ‘UK Internal Market Bill: Legislative Consent’ (November 2020).

55 Addressing the Scottish Parliament, Mike Russell MSP condemned the UK government’s proposals as ‘a nakedly political ploy—a predetermined draconian solution in search of a non-existent problem’: Constitution and Cabinet Directorate (n 54) 6.

56 At Union level, Member State governments are represented alongside the Commission and European Parliament through the Council of the European Union. See eg art 16 TEU and art 294 TFEU.

57 The duality of the UK government’s role as sub-national and UK regulator is also visible in relation to the COVID-19 pandemic response. See GP Evans, ‘Devolution and COVID-19: Towards a “New Normal” in the Territorial Constitution?’ [2021] PL 19, 26.

58 For discussion, see eg Jim Gallagher, ‘The Ghost in the Machine? The Government of England’ in Michael Kenny et al (eds), *Governing England: English Identity and Institutions in a Changing United Kingdom* (OUP 2018).

59 The government’s power is not unlimited: the exercise of its delegated powers remains subject to parliamentary approval through the affirmative resolution procedure. See eg UKIMA, s 6(6) and s 8(8).

60 In relation to the scope of the non-discrimination principle in the field of goods, see eg ibid s 6(5) and s 8(7).

61 See eg ibid ss 6(7)–(10).

62 ibid s 10(3).

63 I am grateful to the anonymous reviewer for raising this point.

64 Where it exists, scrutiny is provided principally by the UK Parliament through the requirement to comply with the affirmative resolution procedure. See eg UKIMA, ss 6(6) and 8(8).

65 Evidence of Mike Russell MSP, House of Lords Common Frameworks Scrutiny Committee, *Common Frameworks: Building a Cooperative Union* (HL 259, 2021) 37.

66 For analysis, see eg Tierney (n 49).

67 See n 36 above.

68 On historical aversion to federalism in UK constitutional discourse, see eg D Armitage, ‘Conclusion: We Have Always Been Federal’ in Schütze and Tierney (n 39).

69 I am grateful to Neil Walker for this point. See also D Wincott, C Murray and G Davies, ‘The Anglo-British Imaginary and the Rebuilding of the UK’s Territorial Constitution after Brexit: Unitary State or Union State’ [2021] Territory, Politics, Governance <www.tandfonline.com/doi/pdf/10.1080/21622671.2021.1921613>.

70 For a broader perspective on the UK’s material constitution referencing additional considerations of political unity, institutions and social relations, see M Goldoni and MA Wilkinson, ‘The Material Constitution’ (2018) 81 MLR 567.

71 Nicolas Barber, *The Principles of Constitutionalism* (OUP 2018) 2–6. Constitutions may also discharge further functions, including symbolic ones. See eg James Bryce, *Constitutions* (OUP 1905) 158. In relation to the UK, see Michael Gordon, *Parliamentary Sovereignty in the UK Constitution: Process, Politics and Democracy* (Hart Publishing 2015) 25–8.

72 See generally Bryce (n 71) vii; KC Wheare, *Modern Constitutions* (OUP 1975) 1; C McIlwain, *Constitutionalism: Ancient and Modern* (Liberty Press 1947). See also House of Lords Constitution Committee, *Reviewing the Constitution: Terms of Reference and Method of Working. First Report* (HL 11, 2001) 20.

73 Goldoni and Wilkinson ascribe particular weight to such teleological aims as drivers of a constitution’s material identity. See Goldoni and Wilkinson (n 70) 558–9.

74 See eg *Entick v Carrington* (1765) EWHC KB J98. See also David Feldman, *Civil Liberties and Human Rights in England and Wales* (OUP 2002); Stephen Sedley, *Lions under the Throne: Essays on the History of English Public Law* (OUP 2015).

75 See also N Walker, ‘Our Constitutional Unsettlement’ [2014] PL 529, 533. Of course, the preferences of majority governments may be affected by other political constraints, eg scrutiny in the House of Lords.

76 This point was neatly satirised in the BBC television series, *Yes, Minister*, in which the fictional Permanent Secretary, Sir Humphrey Appleby, quipped: ‘I have served 11 governments in the past 30 years. If I’d believed in all their policies, I’d have been passionately committed to keeping out of the Common Market, and passionately committed to joining it. I’d have been utterly convinced of the rightness of nationalising steel and of denationalising it and renationalising it … I’d have been a Keynesian and a Friedmanite, a grammar school preserver and destroyer, a nationalisation freak and a privatisation maniac, but above all, I would have been a stark-staring raving schizophrenic’.

77 See eg Aneurin Bevan, *In Place of Fear* (reprint, Aneurin Bevan Society 2008) 100; S Cripps, ‘Can Socialism Come by Constitutional Methods?’ in Christopher Addison and others, *Problems of a Socialist Government* (Gollancz 1933) 35; more recently, Richard Bellamy, *Political Constitutionalism: A Republican Defence of the Constitutionality of Democracy* (CUP 2007).

78 See eg Thomas Horsley, *The European Court of Justice as an Institutional Actor: Judicial Policymaking and its Limits* (CUP 2018); Michael Wilkinson, *Authoritarian Liberalism and the Transformation of Modern Europe* (OUP 2021).

79 Classically, *Van Gend en Loos* (n 6).

80 See eg E Stein, ‘Lawyers, Judges, and the Making of a Transnational Constitution’ (1981) 75 AJIL 1; JHH Weiler, ‘The Transformation of Europe’ (1991) 100 Yale LJ 2403.

81 On horizontal direct effect in EU free movement law, see eg S Löwisch, *Die Horizontale Direktwirkung der Europäischen Grundfreiheite*n (Nomos 2009).

82 See eg Case C-201/15 *AGET Iraklis* [2016] ECLI:EU:C:2016:972, para 48; Case C-140/03 *Commission v Greece* [2005] EU:C:2005:242, para 27.

83 See generally eg Panos Koutrakos, Niamh Nic Shuibhne and Phil Syrpis, *Exceptions from EU Free Movement Law Derogation, Justification, and Proportionality* (Hart Publishing 2016). For a broader critical perspective, see also Grimm (n 10) and Wilkinson (n 78).

84 Alcohol (Minimum Pricing) (Scotland) Act 2012; Case C-333/14 *Scotch Whisky Association* ECLI:EU:C:2015:845.

85 UKIMA, ss 4 and 9.

86 See eg Case 302/86 *Commission v Denmark* ECLI:EU:C:1988:421; *Schmidberger* (n 10); Case C-320/03 *Commission v Austria* ECLI:EU:C:2005:684; Case C-441/04 *A-Punkt Schmuckhandels GmbH v Schmidt* ECLI:EU:C:2006:141.

87 See section 4B above.

88 For criticism in relation to EU law, see Scottish government, ‘After BREXIT: The UK Internal Market Act & Devolution’ (March 2021) 17.

89 Contrast here compliance with rights under the European Convention on Human Rights. See eg Scotland Act 1998, s 29(2)(b). See also s 30A in relation to the modification of retained EU law.

90 Part 4 of the UKIMA empowers the devolved administrations (and UK government) to request independent technical advice from the OIM on the economic effects of proposed regulations on the functioning of the UK internal market. See s 34.

91 See here especially the Welsh government, which has sought judicial review of the 2020 Act. At the time of writing, the Welsh government has been refused to grant permission for judicial review pending its enactment of specific legislation. See *R (on the Application of the Counsel General for Wales) v Secretary of State for Business, Energy and Industrial Strategy* [2022] EWCA Civ 118.

92 Equality Act 2010, s 149.

93 JAG Griffith, ‘The Political Constitution’ (1979) 42 MLR 1.

94 See eg Human Rights Act 1998, ss 3 and 4; Scotland Act 1998, s 29; Government of Wales Act 2006, s 94. For analysis, see eg C McCorkindale, A McHarg and P Scott, ‘The Courts, Devolution, and Constitutional Review’ (2017) University of Queensland Law Review 289.

95 See, *obiter*, Lord Steyn in *Jackson v Attorney General* [2005] UKHL 56, [2006] 1 AC 262 [102]. Thereafter, see Lord Hope in *AXA General Insurance Ltd and Others v The Lord Advocate and Others (Scotland)* [2011] UKSC 46, [2012] 1 AC 868 [50]–[51]. See also Lord Mance and Lord Neuberger in *HS2 Action Alliance Ltd v Secretary of State for Transport* [2014] UKSC 3, [2014] 1 WLR 324 [207]; Lord Carnwath in *R (on the application of Privacy International) v Investigatory Powers Tribunal and Others* [2019] UKSC 22, [2019] 2 WLR 1219 [123] and [131]. This development aligns with the normative world of common law constitutionalism. See eg Trevor Allan, *Law, Liberty, and Justice: The Legal Foundations of British Constitutionalism* (Clarendon Press 1993); Trevor Allan, *The Sovereignty of Law: Freedom, Constitution, and Common* Law (OUP 2013); J Laws, ‘Law and Democracy’ [1995] PL 72.

96 In relation to goods, UKIMA, s 1(3) outlines expressly that the direct effect of the market access principles is limited to the terms of the UKIMA. It is therefore not, as in EU law, to be construed independently as a general principle of broader application.

97 See eg Scotland Act 1998, s 29(2)(d).

98 See I Mahler, ‘National Courts as European Community Courts’ (1994) 14 LS 226.

99 See UKIMA, sch 1.

100 In EU internal market law, that category of exceptions has its origins in *Cassis de Dijon* (n 29). For criticism, see eg Scottish government (n 88) 7.

101 See eg UKIMA, ss 6(6) and 8(8) discussed in section 4B above.

102 William Cash (Conservative), HC Deb 17 January 2018, vol 634, col 933.

103 Of course, Parliament may override adverse judicial decisions in accordance with the doctrine of parliamentary sovereignty. See section 5B above.

104
*R (on the application of Miller and another) v Secretary of State for Exiting the European Union* [2017] UKSC 5, [2018] AC 61; *R (on the application of Miller) v The Prime Minister* [2019] UKSC 41, [2019] WLR 589.

105 See eg *The Independent Review of Administrative Law* (CP 407, 2021); Judicial Review and Courts Act 2022.

106 See eg R Ekins and G Gee, ‘Putting Judicial Power in its Place’ (2018) 36 UQLJ 375. Contrast P Craig, ‘Judicial Power the Judicial Power Project and the UK’ (2018) 36 UQLJ 355.

107 See *Overview of the UK Internal Market* (OIM 6, 2022).

108 See eg *Counsel General for Wales v Secretary of State for Business, Energy and Industrial Strategy* (n 91) [12].

109 On progress, see Cabinet Office, ‘Government Response to the House of Lords Common Frameworks Scrutiny Committee Report: Common Frameworks: Building a Cooperative Union’ (May 2021).

110 See esp UKIMA, s 36.

## Notes

I wish to thank Mike Gordon, Neil Walker and the anonymous reviewers for their helpful comments on earlier versions of this article. I am also grateful for feedback I received at presentations delivered at the Wales Governance Centre, University of Cardiff (28 October 2021) and as a Research Fellow at the Israeli Institute for Advanced Studies (2019–20). All errors remain my own.

